# Supplementary material for: α-Synuclein Preformed Fibrils Bind to β-Neurexins and Impair β-Neurexin-Mediated Presynaptic Organization
Source: Cells. 2023 Apr 4;12(7):1083. doi: 10.3390/cells12071083 (PMC10093570; doi:10.3390/cells12071083)
Supplement: Supplementary file 1 [file cells-12-01083-s001.zip › cells-2283425-supplementary.pdf]

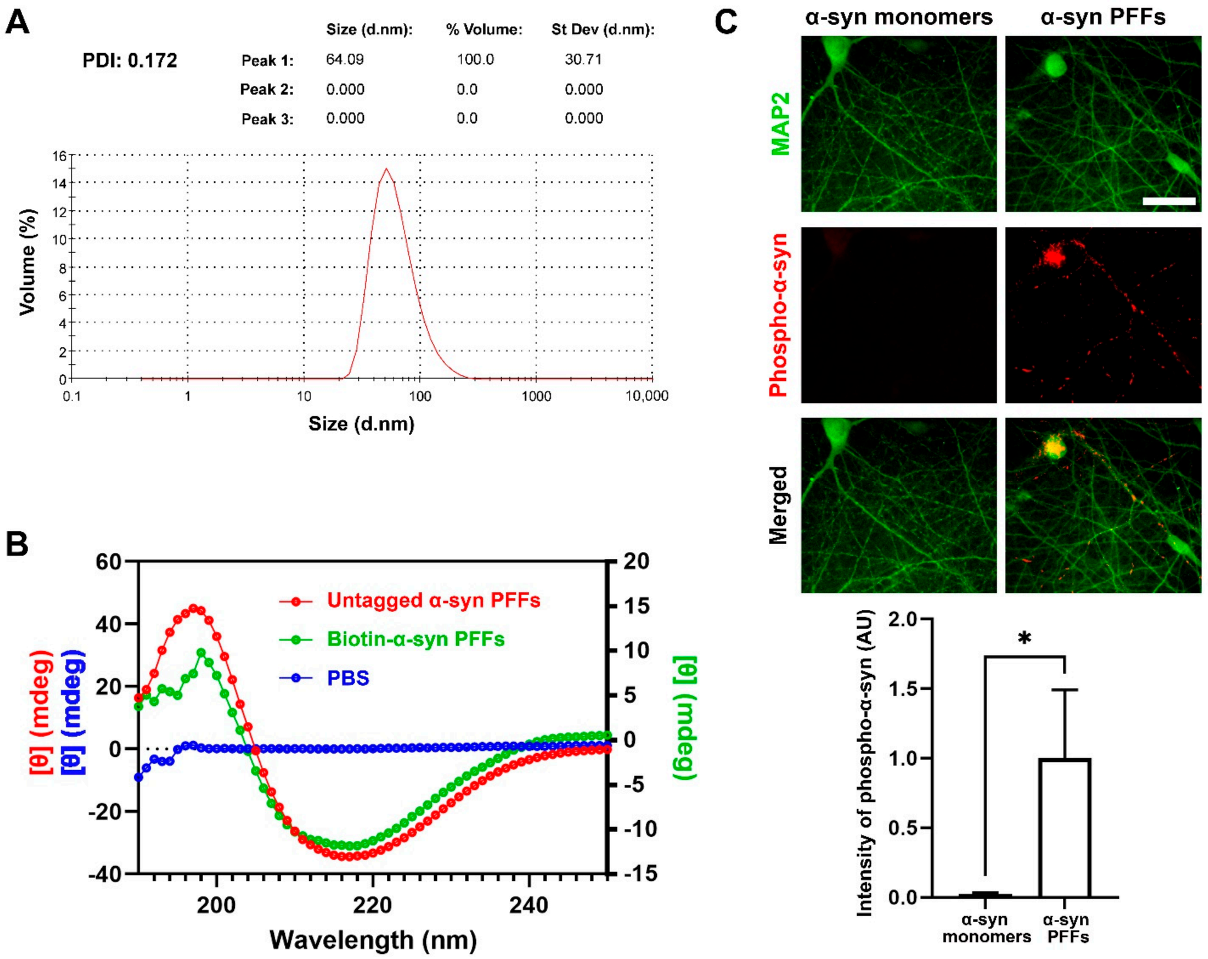

Supplementary Figure S1. The biotinylation of α-syn PFFs has no significant effect on their overall size, secondary structure or capability to induce hyperphosphorylation of endogenous α-synuclein in neurons. (A) Dynamic light scattering analysis of biotin-α-syn PFFs confirming that their average size is similar to that of untagged α-syn PFFs (64.09 nm vs 52.85 nm) and that there is a single peak with polydispersity index (PDI) of 0.172, suggesting that they are relatively homogeneous aggregates like untagged α-syn PFFs. **(B)** Circular dichroism (CD) spectra of PBS (blue), untagged α-syn PFFs (0.25 mg/mL; red) or biotin-α-syn PFFs (0.15 mg/mL; green). A positive peak at around 195 – 200 nm followed by a negative peak at around 210 – 220 nm is a typical spectrum pattern of β-sheet structures [**88**]. The CD spectrum pattern of biotin-α-syn PFFs (green) is very similar to that of untagged α-syn PFFs (red), suggesting that biotinylation does not affect the secondary structure of α-syn PFFs. **(C)** Biotin-α-syn PFFs can induce hyperphosphorylation of endogenous α-synuclein in hippocampal neuron cultures. Hippocampal cultured neurons were treated with biotin-α-syn PFFs (2.5 μg/ml [174 nM monomer equivalent]) or biotin-α-syn monomers (2.5 μg/ml) at 7 days *in vitro* (DIV). The cultures were maintained until 14 DIV and then immunostained for phospho-α-synuclein (red) and MAP2 (green) as a dendrite marker. Similar to untagged α-syn PFFs (Figure 1D), treatment with biotin-α-syn PFFs, but not biotin-α-syn monomers, induced clusters of phosphorylated α-synuclein immunoreactivity along neurites (**middle right panel**, red). Bar graph (**bottom**) showing the intensity quantification of phospho-α-synuclein immunoreactivity in neurons treated with biotin-α-syn monomers or biotin-α-syn PFFs. Mann-Whitney U test, *$P < 0.05$. Data are presented as mean ± SEM. (n = 3 and 5 images for biotin-α-syn monomers or biotin-α-syn PFFs, respectively, from one experiment). Scale bar: 30 μm.

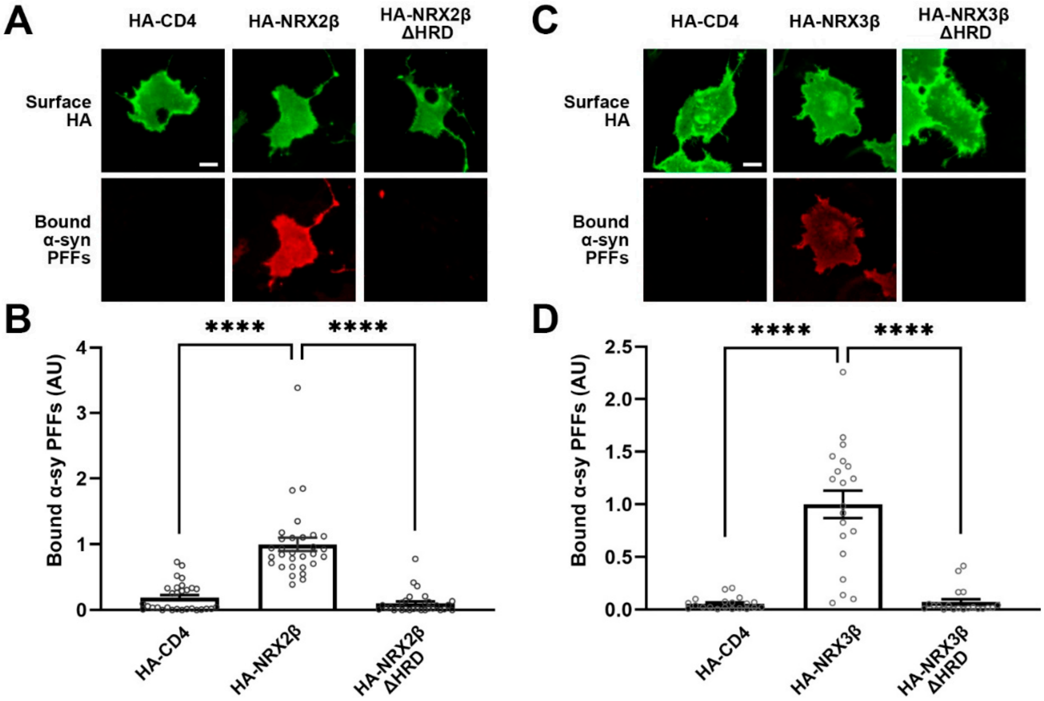

Supplementary Figure S2. The N-terminal histidine-rich domain of neurexin 2β and 3β is responsible for the binding of α-syn PFFs. (**A, C**) Representative images showing the binding of 1 μM biotin-α-syn PFFs to COS-7 cells expressing the indicated HA-NRX2β (**A**) and HA-NRX3β (**C**) constructs, either full-length or lacking the histidine-rich domain (HRD) (ΔHRD), or HA-CD4, a negative control. COS-7 cells expressing HA-NRX2β ΔHRD or HA-NRX3β ΔHRD show no binding of biotin-α-syn PFFs, appearing comparable to those expressing HA-CD4. All constructs have significant surface HA expression. (**B, D**) Quantification of the average intensity of bound biotin-α-syn PFFs on COS-7 cells expressing the indicated HA-NRX2β/3β constructs or HA-CD4. Kruskal-Wallis one-way ANOVA, $P < 0.0001$. ****$P < 0.0001$ in the indicated comparisons by Dunn's multiple comparisons test. N.S., not significant. Data are presented as mean ± SEM. (n = 30 and 20 cells for **B and D** from three and two independent experiments, respectively).

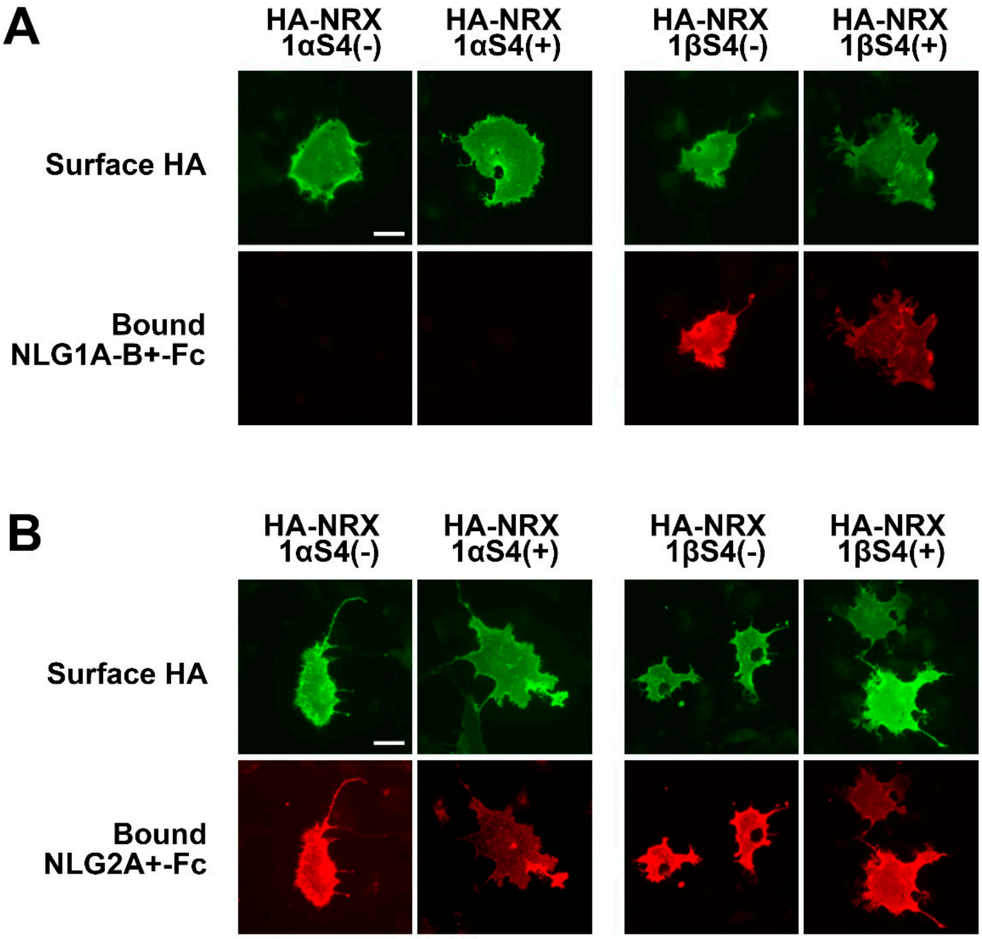

Supplementary Figure S3. NLG1A-B+ binds to NRX1β but not NRX1α whereas NLG2A+ binds to both NRX1α and NRX1β. (**A, B**) Representative images showing the binding of NLG1A-B+-Fc (**A**) and NLG2A+-Fc (**B**) to COS-7 cells expressing the indicated HA-NRXs constructs. As reported previously [22,62], NLG1A-B+-Fc binds to only NRX1β, and not NRX1α. In contrast, NLG2A+-Fc binds to both NRX1α and 1β. Scale bars: 30 μm.

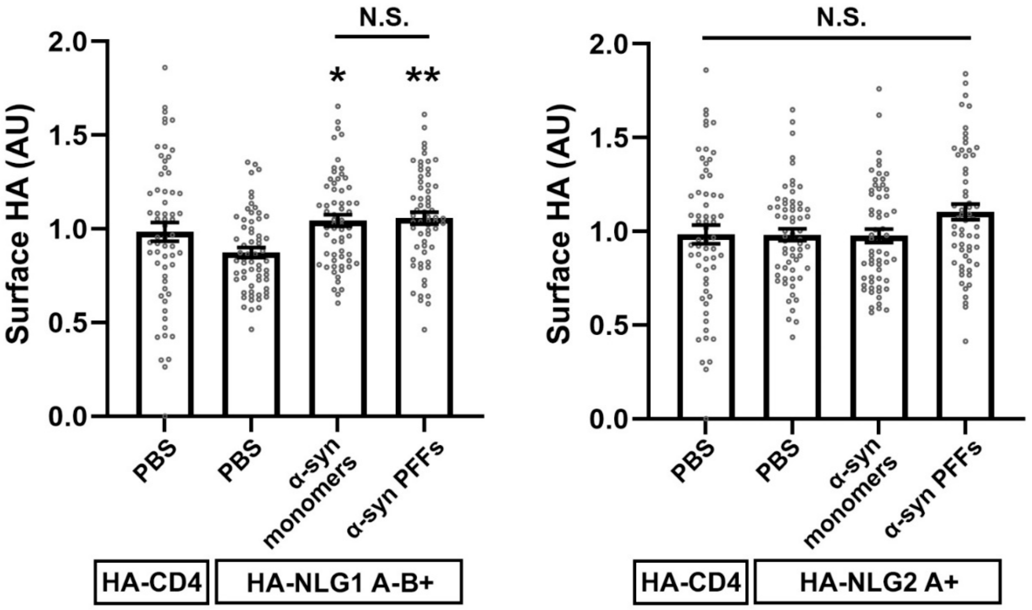

Supplementary Figure S4. α-syn PFF treatment does not decrease surface expression of HA-NLG1A-B+ or HA-NLG2A+ on HEK293T cells in the co-culture-based artificial synapse formation assays presented in Figure 7. Quantification of surface HA expression level of HA-NLG1A-B+ (left) and HA-NLG2A+ (right) under the indicated treatment conditions in the co-culture-based artificial synapse formation assays presented in **Figure 7**. The level of surface HA expression after α-syn PFF treatment in the NLG1A-B+ group (left) does not correlate with the NLG1A-B+-induced VGLUT1 and VGAT accumulation phenotype after α-syn PFF treatment (**Fig. 7B and C**), excluding the possibility that the phenotype is due to decreased surface expression of HA-NLG1A-B+ on HEK293T cells after α-syn PFF treatment. Kruskal-Wallis one-way ANOVA, $P < 0.001$ for HA-NLG1 and $P = 0.1439$ for HA-NLG2A+. *$P < 0.01$ and **$P < 0.001$ for the indicated comparisons by Dunn's multiple comparisons test. N.S., not significant. Data are presented as mean ± SEM. (n > 60 cells for each from four independent experiments).

References in Supplementary figure legends

22. Craig, A.M.; Kang, Y. Neurexin-neuroligin signaling in synapse development. Curr Opin Neurobiol **2007**, 17, 43-52, doi:S0959-4388(07)00013-X [pii]10.1016/j.conb.2007.01.011.

62. Boucard, A.A.; Chubykin, A.A.; Comoletti, D.; Taylor, P.; Sudhof, T.C. A splice code for trans-synaptic cell adhesion mediated by binding of neuroligin 1 to alpha- and beta-neurexins. Neuron **2005**, 48, 229-236, doi:10.1016/j.neuron.2005.08.026.

88. Kelly, S.M.; Price, N.C. The use of circular dichroism in the investigation of protein structure and function. Curr Protein Pept Sci **2000**, 1, 349-384, doi:10.2174/1389203003381315.
